# Enlisting in the army in the Jewish Ultraorthodox community and the consequences for wellbeing

**DOI:** 10.3389/fpsyg.2023.1132624

**Published:** 2023-05-12

**Authors:** Nechumi Malovicki-Yaffe, Yael Itzhaki-Braun, Shomi Shahar-Rosenblum

**Affiliations:** ^1^Department of Public Policy, Gerson H. Gordon Faculty of Social Science, Tel Aviv University, Tel Aviv, Israel; ^2^Time Data Institute, Jerusalem, Israel

**Keywords:** Ultraorthodox, military service, wellbeing, societal conditional regard, stigma

## Abstract

Israeli law requires citizens to enlist in the army at 18 years old. However, the Jewish Ultraorthodox community has a longstanding agreement with the state that members of this community will not have to enlist in the army, given its spiritual leaders’ strong opposition. Still, there are young men who go against the communal norms and enlist. In the current study we investigated these young men and the contribution of their self-esteem (a personal resource), their sense of community (a communal resource), and the community members’ attitudes toward them (societal conditional regard, both positive and negative, and stigma) to their wellbeing. The current study consisted of 153 participants between the ages of 20 and 55 (*M* = 29.64, SD = 6.89). A path analysis model indicated the protective role played by self-esteem and sense of community in participants’ wellbeing, and the risk factor posed by societal conditional negative regard and stigma. Moreover, self-esteem was found to mediate between income and wellbeing, whereas sense of community was found to mediate between societal conditional negative regard and wellbeing, and between stigma and wellbeing. The discussion highlights the complexity of the protective role played by sense of community against the risk of experiencing societal conditional negative regard and stigma. It also addresses the importance of promoting intervention programs during the army service of these young men, with a focus on promoting their self-esteem and on the presence of a spiritual leadership that legitimizes working, serving in the army, and yet still being part of the community.

## Public policy relevance statement

Although enlisting in the Israeli army is enshrined in law, for young men from the Ultraorthodox community, army enlistment is considered to be a violation of the communal norms and culture. In the current study we suggest that young men from this community who enlist are forced to cope with stigma and negative attitudes from the community, impairing their wellbeing and potentially hindering their proper integration into adulthood. Thus, the state, together with spiritual leaders and professionals in NGOs who treat these young boys, should examine how to open the lines of communication between the military and the Ultraorthodox community in order to reduce these negative attitudes. They should also find ways to enable these young men to remain part of the Ultraorthodox community, despite their military service.

## Introduction

Military service marks the transition to adulthood for many young people. This is especially the case in countries in which military service is mandatory (Dar and Kimhi, 2001; Kelty et al., 2010). This stage is considered to be complex, as young people must begin managing independently and making decisions regarding their future studies and careers. For at-risk populations, this stage is even more complex due to the lack of resources that generally typifies these populations (Sulimani-Aidan, 2017). As wellbeing is a key factor for success in the transition to adulthood (Lamborn and Groh, 2009), it is crucial to recognize the risk and protective factors for wellbeing among such populations at this life-defining stage. Various factors can create risk situations for young people at this time, including poverty, family difficulties, etc. (Osgood et al., 2005). For young people who belong to the Ultraorthodox Jewish community in Israel and have enlisted in the army, a unique risk factor exists. Given that the Ultraorthodox community opposes army enlistment, those who do enlist actually violate communal norms. The violation of such norms in Ultraorthodox society has social consequences that have been found in various studies to harm the wellbeing of those who do so (Itzhaki et al., 2020; Itzhaki-Braun and Sulimani-Aidan, 2022).

In the current study we explored the wellbeing of young men who grew up in the Ultraorthodox community in Israel and violated communal norms by enlisting in the Israel Defense Forces (IDF). To the best of our knowledge, the current study is the first to focus on this very unique group. Typically, young adults in Israel who complete their military service return to their families and communities and start their adult lives, working and/or studying. Thus, they are considered to be a normative group and as such do not receive much attention in the scientific literature. However, those from the Ultraorthodox community must return to these communities after they have violated the communal norms (i.e., enlisting in the army) and must bear the consequences of this choice – consequences that can harm their wellbeing. Specifically, we examined the joint contribution to their wellbeing (i.e., the wellbeing of young men from the Ultraorthodox community who completed their military service) of the community’s perceptions (stigma) and attitudes [societal conditional regard (SCR)] toward them, as experienced by these men. In addition, we examined the mediating role of personal (self-esteem) and communal (sense of community) resources, in this association. The present study offers a glimpse into the phenomenon of religious minority community members who depart from established norms, and examines the potential implications of such deviations for their overall wellbeing. The study provides us with a further understanding of the dynamics of community membership and of the variables that are associated with young men’s psychological outcomes. It also provides a look at the complexity of deviating from norms within religious communities.

### Deviation from social norms

Community life, much like any social group life, is organized around rules and accepted social norms. Social norms are “a generally accepted way of thinking, feeling, or behaving that is endorsed and expected because it is perceived as the right and proper thing to do. It is a rule, value or standard shared by the members of a social group that prescribes appropriate, expected or desirable attitudes and conduct in matters relevant to the group” (Turner, 1991, p. 3). Group norms are further defined as “regularities in attitudes and behavior that characterize a social group and differentiate it from other social groups” (Hogg and Reid, 2006, p. 7). Norms are therefore agreed-upon attitudes, thoughts, and values, alluding to “desirable behaviors” or what one “ought” to do, and relate to the moral obligations prescribed within a specific group (Cialdini and Trost, 1998). Social norms are conveyed by what people do and say in their everyday lives. These norms can be conveyed indirectly (e.g., inferring norms from others’ behaviors) but also directly (e.g., intentionally talking about what is and is not normative of the group) (Hogg and Reid, 2006).

Deviating from social norms often bears a social punishment, such as a decline in social status or exclusion, particularly if the social norms are important to the group (Festinger, 1950). There is no question that social norms are the primary source of social order (Feld, 2002). Individuals who defy the social norms affect other people’s perceptions of norms in their community which, in turn, may reshape collective behaviors (e.g., Paluck and Shepherd, 2012). Research has shown that religion is one of the frameworks that creates rigidity in relation to maintaining norms both at the community level and at the individual level (Tittle and Welch, 1983; Johnson et al., 2012). Leach and Gore (2022) found that it was not just religion itself that predicted intolerance of deviating behaviors. Rather, individuals with a highly orthodox orientation toward religion have stricter rules and punishments and provide little leeway when it comes to norm violations. As such, there is a high intolerance of deviating behavior in religious communities. Religious communities are quicker to stigmatize individuals who deviate from group norms. Shapiro (2013) examined attitudes toward the LGBT individuals and norms-breaking in the Orthodox and Ultraorthodox Jewish community and found a higher intolerance for change and a greater likelihood for punishing such changes in communities where more traditional practices are maintained, such as in the Orthodox and Ultraorthodox world. Similarly, Vega (2016) conducted a study involving in-depth interviews with 15 men from the Ultraorthodox community and examined the various challenges they faced from their families and community due to their deviation from established norms, such as pursuing military service or higher education. The current study expands this research by exploring and establishing the existence of stigma and SCR, in this context, and examining the potential correlation between these components and overall wellbeing.

### The Ultraorthodox community in Israel

The Ultraorthodox community is a modern construal that emerged in the 18th century as a response to the Enlightenment, which drove Jews away from religion. In an attempt to stop this trend, rabbis encouraged people to withdraw from the secular world as a strategy against modernization, building “walls of holiness” between themselves and the secular world (Bacon, 1991; Hildesheimer, 1994; Katz, 2000; Schreiber, 2002; Feiner, 2011). This segregated lifestyle encompassed all aspects of life, from the formation of a new ideology to segregated residential neighborhoods, separate school systems, and a specific dress code (Friedman and Shilhav, 1986; Shilhav and Friedman, 1989; Stadler, 2002). The Ultraorthodox community that was established in Israel maintained this spirit of isolation, putting “walls of holiness” between them and the rest of the Israeli population. Their ideological struggle with the secular world still exists (Brown, 2000, 2014). This population comprises 12.6% of Israel’s total population (Malach and Cahaner, 2020), and with an average of five children per family (Israel Central Bureau of Statistics, 2020), they are considered to be one of Israel’s poorest sectors (Kliner-Kasir and Tsachor-Shai, 2017; Malovicki-Yaffe et al., 2018a).

A key feature of the Ultraorthodox community in Israel is its being a “society of learners.” Most of the men in the community invest many years in studying sacred Jewish texts, and their wives are the main breadwinners (Stadler, 2009; Malovicki-Yaffe et al., 2018b; Malovicki-Yaffe, 2020). The “society of learners” phenomenon came about as a result of a few social forces (Frankel, 1994; Spiegel, 2011; Leon, 2017; Malechi, 2017). The first was the response to the destruction of religious knowledge during the Holocaust and an attempt to rehabilitate the world of Torah study after the Holocaust. A concerted effort was made to restore this knowledge and to renew the community’s connection to it. As part of this movement, men were actively encouraged to dedicate their lives to the study of the Torah, with the expectation that they would eschew worldly pursuits in order to focus on this sacred endeavor (Friedman, 1991; Brown, 2000; Stadler, 2009; Spiegel, 2011). This learning effort was further reinforced in response to the complicated constitutional legislation concerning army recruitment (Jobani, 2009; Jobani and Perez, 2014).

Military service in Israel is mandatory for all Jewish Israelis between the ages of 18 and 27. Arrangements enabling members of the Ultraorthodox community to avoid the draft were instituted during the first few years of statehood and were conditioned on men being Torah students, meaning they were prohibited from entering the labor market, what become known as “Toratam umanutam” (Frankel, 1994; Spiegel, 2011; Leon, 2017; Malechi, 2017).

Ever since the initial exemption in 1948 (which was originally issued for only 400 students, a number that has increased exponentially), this issue has been the focus of an ongoing rift between Israeli society and the Ultraorthodox community, and it is a topic of major importance in every election cycle (Shilhav and Friedman, 1989; Evans, 2011; Cahaner and Mansfeld, 2012; Ellenson, 2018).

The Ultraorthodox resistance to army enlistment lies in the understanding that serving in the IDF will inevitably lead to a loss of rabbinic authority over these young men, and a swift drift away from religious life. Indeed, enlisting in the army is, among Ultraorthodox boys, often the first step in their becoming secular (Velan et al., 2022). Thus, all Ultraorthodox boys are compelled to learn Torah in the yeshiva from a young age until the time they get married (Finkelman, 2011) so as to mold their behavior and keep them in line with the norms and values of Ultraorthodox Judaism (Itzhaki et al., 2018b). Dropping out of yeshiva is perceived as showing disregard for, or even negating, basic familial values and communal norms (Finkelman, 2011). Given the community’s stance regarding army service – and its ideological conflict with the secular world around it – enlisting is considered to be a violation of communal norms. As a result, those dropouts who join the military become a particularly stigmatized group (Vistoch, 2022). Professionals, who work in NGOs that treat these young boys, have reported that such individuals often have difficulty finding a spouse from the Ultraorthodox community and have very limited job options within the community. Having not acquired a high school education, their ability to integrate into society at large is also limited. The rabbis’ apprehension regarding military service and secularism prompted the formulation of an ideology that encouraged men to remain in yeshiva for extended periods of time while not working (Berman and Klinov, 1997; Gottlieb, 2007). This situation was further compounded by legal limitations which precluded men from entering the labor market unless they had served in the army, resulting in widespread destitution and hardship. At present, approximately 62% of Ultraorthodox households live below the poverty line, and roughly 60% of Ultraorthodox children experience poverty, in stark contrast to the 25% of their non-Ultraorthodox counterparts (Malach et al., 2022). For all of these reasons this group has become a marginalized group in Israeli society, with the consequences of their choice to enlist potentially impairing their wellbeing.

### Wellbeing

Wellbeing can be defined as the fulfillment of mental, physical, and social health needs, and not just as the absence of distress. Wellbeing is subjective and is related to the experience of high levels of positive feeling (Diener and Suh, 2003). One-way people achieve wellbeing is through the building of relationships and the establishment of mutual positive connections between themselves and their environment – that is, family, friends, school, community, and more (Davidson-Arad and Klein, 2011; Jose et al., 2012). Thus, for example, dropping out of school and “cutting this link in the chain” has been found to negatively affect wellbeing (South et al., 2007; Etzion and Romi, 2015). A study that was conducted among 261 (male) Ultraorthodox school dropouts revealed that the unraveling of these young men’s social fabric was rooted in the nature of their relationships with their parents and community. More specifically, relationships with parents and community members were found to be the greatest predictor for their dropping out of school; dropping out of school diminished their involvement with their friends and caused them to lose their sense of community; and all of the aforementioned negatively affected their wellbeing (Itzhaki et al., 2018a,^2020^). Similarly, it has been found in numerous studies that leaving religion and being rejected by one’s community and friends affects wellbeing (Hookway and Habibis, 2015; Ransom et al., 2021; Thoma et al., 2022). Young Ultraorthodox men enlisting in the army are viewed as being on their way toward becoming less religious (Velan et al., 2022) and are exposed to harsh treatment from family and community because they are seen as defying community norms (Vega, 2016).

### The community attitude toward deviation

The greatest punishment a community can inflict is stigmatizing those who deviate from the communal norms (Frable et al., 1990; Crocker et al., 1998; Major and O’Brien, 2005; Jetten and Hornsey, 2010; Monin and O’Connor, 2011; Levine and Marques, 2016). Specifically, the Ultraorthodox community stigmatizes individuals who engage in activities such as using drugs (Loewenthal, 2014) or defining themselves as LGBT – that is, activities/notions that violate communal norms.

In Link and Phelan’s (2001) model of stigma, when a person is labeled as different, this label is then cognitively linked to negative stereotypes embedded in cultural beliefs. The affected individuals may subsequently experience loss of status and discrimination, which can result in poor outcomes vis-à-vis prior social, economic, and political power dynamics (Becker et al., 2019). Stigma is usually studied in regard to a certain characteristic that globally sets a group apart from the rest of society, such as race, gender, sexual orientation, religion, age, disability, and socioeconomic status (e.g., Blank et al., 2002, 2013; Shamsalinia et al., 2019). However, discrete stigmatized groups can be found within religious communities. In these communities, norms are very strict and explicit, and they encompass a strict dress code for men and women, and a prescribed set of daily behaviors and routines (Davidman, 1991; Valins, 2000). There are groups that are not globally stigmatized, but become a group marked by stigma because of behavior that is inconsistent with the norms of the religious community.

Another way communities control their members is by conditioning their affection toward community members: a notion better known as SCR. SCR is a new theoretical concept developed in recent years to understand the attitude of the religious community toward members who violate community norms (Itzhaki et al., 2018a). SCR regard is based on self-determination theory (SDT; Deci and Ryan, 1985), and it focuses on community members’ attitudes as experienced by individuals inside the community. It connotes a situation in which the granting of a society’s warmth and affection is contingent upon the individual’s behaving in accordance with the society’s expectations. In other words, it could be said that SCR is the community’s way of controlling and measuring an individual’s degree of conformity to its norms. In the context of societal conditional *positive* regard (SCPR), community members provide more attention, affection, and appreciation than usual when the individual meets their expectations. In the context of societal conditional *negative* regard (SCNR), community members provide less affection, warmth, and appreciation than usual when the individual does not meet their expectations. Itzhaki et al. (2018b) found that dropping out of the communal educational framework was considered to be a norms violation in the Ultraorthodox community, but when dropout youths aimed to conform in other ways – for instance, dressing in ways deemed acceptable – people in the community gave them more attention than they otherwise would have. However, when they did not do so, people in the community gave them less love and affection than they otherwise would have. Given that SCR is a religious community’s psychological-emotional way of making its members behave in accordance with its norms, we wished to examine whether it could be a significant factor in the wellbeing of Ultraorthodox youth who enlist in the army (Itzhaki et al., 2018a). The concept of SCR is based on parental conditional regard (PCR), which is a construct referring to the granting of parental love and acceptance contingent upon children’s being compliant with their parents’ expectations. According to PCR, both parental conditional positive regard (PCPR) and parental conditional negative regard (PCNR) are considered to be practices that suppress autonomy, and can have many negative effects such as feelings of internal compulsion, self-aggrandizement following success, self-devaluation, shame following failure, lower levels of self-esteem, and lower levels of sense of community (Assor et al., 2004; Roth et al., 2009; Assor and Tal, 2012; Itzhaki et al., 2018b; Sabag, 2022). Due to these effects, many studies have examined the role of these effects as mediators in the correlation between PCR and psychological outcomes such as wellbeing (Perrone et al., 2016; Curran, 2018; Sabag, 2022). Based on these studies, we found it crucial to examine the role of self-esteem and sense of community as mediators in the association between the community’s attitude toward individuals who completed military service and these individuals’ wellbeing. Moreover, as individuals’ personal and social resources have been found to mediate the relationship between stigma and psychological outcomes such as depression, anxiety, and wellbeing (Hatzenbuehler, 2009; Caserta et al., 2016; Turan et al., 2019), we examined the role of self-esteem and sense of community as mediators for the correlation between stigma and wellbeing as well.

### Personal and communal resources

In terms of personal resources, we looked at self-esteem, which is usually reinforced by community membership. A systematic review of nearly 800 studies has led to the conclusion that religious thought and practice contribute beneficially to multiple behavioral and health-related outcomes concerning, among other things, self-esteem, and wellbeing (Johnson et al., 2002). Regnerus and Elder (2003) found that religious organizations and communities promoted the building of qualities such as self-esteem and mastery among young people by providing them with opportunities for positive reflected appraisals (e.g., during group activities) and by fostering the growth of spiritual resources (e.g., faith and hope) (Jackson and Bergeman, 2011). In addition, high levels of self-esteem have been found to make a positive contribution to wellbeing among at-risk religious youth (Itzhaki et al., 2018a).

In terms of communal resources, we examined individuals’ sense of community. Sense of community is a concept which has been defined as individuals’ emotional connection, friendship, and sense of belonging within a given community, as well as their ability to influence other members of the community, and the belief that the needs of the individual will be addressed because the person is part of the community (McMillan and Chavis, 1986; Braun-Lewensohn et al., 2013). Research has shown that people from religious communities are more likely to experience a sense of community (Stroope, 2011) and benefit from this sense of community; namely, sense of community has been found to be a protective factor against negative psychological outcomes and to lead to higher levels of wellbeing (Lim and Putnam, 2010; Braun-Lewensohn et al., 2013). A recent study among the Ultraorthodox community found that sense of community was positively associated with life satisfaction and wellbeing (Russo-Netzer, 2019).

### Study goal

The goal of this study was to examine the implications of community attitudes for the wellbeing of community members of religious minorities who deviate from the norms of their community. To do so, we examined the contribution of the perception of the community’s attitude, together with personal and communal resources, to the wellbeing of young men who grew up in the Ultraorthodox Jewish community in Israel and completed their military service. Based on the scientific literature we hypothesized that (a) self-esteem, sense of community, and SCPR would contribute to higher levels of wellbeing, whereas stigma and SCNR would contribute to lower levels of wellbeing, and (b) self-esteem and sense of community would mediate the association between stigma and wellbeing, and between SCR and wellbeing.

## Materials and methods

### Participants and procedure

Potential participants were 188 Ultraorthodox men who served in the Israeli army and had finished their service during the previous 5 years. It is important to mention that given the agreement with the state that Ultraorthodox young men are not required to enlist in the army, those who choose to do so can enlist at any age. Thus, we had no criteria regarding age; rather, inclusion criteria regarded family source (growing up in Ultraorthodox home), duration of service (at least a year), visibility of service (i.e., wearing a uniform), and the amount of time that had elapsed since finishing their service (up to 5 years). As a result, 13 individuals were excluded, as they identified as not having grown up in Ultraorthodox homes; 11 were excluded as they served in the army for less than a year; and another 11 were excluded as they did not serve in military uniforms (thereby avoiding the stigma and shame that, in the Ultraorthodox world, accompanies being identified as serving in the military). As such, a total of 153 participants comprised the final analysis sample. Participants’ age ranged between 20 and 55 (*M* = 29.64, SD = 6.89); 56% served in uniform for a year; and 44% served in uniform for more than a year. The average time that had passed since they finished their service was (*M* = 2.7, SD = 1.2). In terms of income, 39% reported earning an above-average salary (vis-a-vis Israeli norms); 17% reported earning an average salary; 35% reported earning a below-average salary; and 9% were missing data. The current study employed a cross-sectional survey. Questionnaires, procedures, consent forms, and instructions were reviewed and approved by the Institutional Review Board (IRB) of the authors’ university.

Given how difficult it is to gain access to members of the Ultraorthodox community, and especially a small invisible sub-group within it, we collaborated with “Netzach Yehuda,” an NGO, that give services of accompaniment and support to the Ultraorthodox battalion within the IDF (Lara and Pabón, 2019). They forwarded our questionnaire to alumni, and we were also granted entry into unique Facebook and WhatsApp groups by friends and personal connections. Participants filled out an online questionnaire and were compensated for their time by taking part in a raffle whose prize was $50.

### Measures

#### Subjective wellbeing

This questionnaire was developed by Bradburn (1969) and assesses the degree of the individual’s current subjective wellbeing. It consists of 10 items such as: “I am satisfied with an accomplishment I achieved.” Participants were asked to rate the extent to which they agreed with each item on a 4-point Likert-type scale, ranging from 1 (*not at all*) to 4 (*totally*). The Cronbach’s alpha reliability in the present study for this measure was α = 0.76.

#### Sense of community

This questionnaire was based on the Winograd-Jean (2005) short version of the Davidson and Cotter’s sense of community questionnaire Davidson and Cotter’s (1986), which includes 10 items such as: “I feel safe in my community.” Participants were asked to rate the extent to which they agreed with each item on a 5-point Likert-type scale ranging from 1 (*strongly disagree)* to 5 *(strongly agree)*. The Cronbach’s alpha reliability in the present study for this measure was α = 0.88.

#### Societal conditional regard

This questionnaire was developed by Winograd-Jean (2005) and was based on the PCR questionnaire, originally developed by Assor et al. (2004). In the current study, SCR was examined in relation to the individual’s observance of the norms of the Ultraorthodox community, as one who completed military service. The questionnaire consisted of 11 items. Five of those items related to SCPR, such as: “If (or when) a person who completed the military service behaves like an Ultraorthodox (praying, setting times to learn Jewish studies, use an Ultraorthodox language), people in the community give him more warmth and affection than usual.” The other six items addressed SCNR, such as: “If (or when) a person who completed the military service hangs out with a partner who is not considered appropriate by the Ultraorthodox community, people in the community give him less warmth and affection than they usually do.” Participants were asked to evaluate their agreement on a 5-point Likert-type scale ranging from 1 *(do not agree at all)* to 5 *(greatly agree)*. The Cronbach’s alpha reliability in the present study for this measure was α = 0.83 for SCPR and α = 0.87 for SCNR.

#### Self-esteem

This questionnaire was developed by Rosenberg (1965) and consists of 10 items such as: “I feel that I’m a person of worth, at least on an equal plane with others.” Participants were asked to rate the extent to which they agreed with each item on a 5-point Likert-type scale, ranging from 1 *(strongly disagree)* to 5 *(strongly agree)*. The Cronbach’s alpha reliability of the questionnaire in the present study for this measure was α = 0.79.

### Perception of stigma

This questionnaire was developed by the authors based on the stigma questionnaire, originally developed by Berger et al. (2001). It consisted of 11 items capturing the stigma in the community toward boys who enlist in the army, with items such as “Most people in the Ultraorthodox community think that an Ultraorthodox guy who enlists in the army is a failure.” Answers were rated on a 5-point Likert-type scale, ranging from 1 *(strongly disagree)* to 5 *(strongly agree)*. The Cronbach’s alpha reliability of the questionnaire in the present study for this measure was α = 0.93.

### Income

Participants read the statement, “An average income for an Ultraorthodox household is 14,750 NIS (5,000$±); what is the income level of your household?” Participants were asked to rate their income on a scale from 1 to 5, with 1 signifying “well above average” to 5 signifying “well below average.”

## Results

Table 1 provides means, standard deviations, and correlations for all of the study variables. Wellbeing was found to be positively correlated with income (*r* = 0.24, *p* = 0.007), self-esteem (*r* = 0.60, *p* = 0.001), and sense of community (*r* = 0.40, *p* = 0.001), and negatively correlated with SCNR (−0.24, *p* = 0.004).

**TABLE 1:** Correlations, means, and standard deviations between the study variables.

Measures	*M*	SD	1	2	3	4	5	6	7	8
1. Age	29.69	6.89	–	0.57***	0.03	0.10	-0.17	0.21*	0.22*	0.14
2. Income	3.11	1.25		–	0.11	0.03	-0.10	0.28**	0.09	0.24**
3. SCPR	3.19	0.87			–	0.61***	0.28***	-0.01	-0.10	0.02
4. SCNR	2.91	0.98				-	0.60***	-0.20*	-0.39***	-0.24***
5. Stigma	2.90	0.99					-	-0.21*	-0.45***	-0.14
6. Self-esteem	4.06	0.64						-	0.32***	0.60***
7. Sense of community	3.27	0.98							-	0.40***
8. Wellbeing	3.79	0.61					-			-

*p < 0.05, **p < 0.01, ***p < 0.001.

To examine the effects of age, income, stigma, SCR, self-esteem, and sense of community on wellbeing, we conducted hierarchical regression, which is displayed in Table 2. In the first step, we inserted the demographic variables of age and income. Income contributed positively to wellbeing (β = 0.32, *p* = 0.009), whereas age made no contribution to wellbeing. This step contributed 8% to the explained variance. In the second model, we inserted the variables that reflect the communal attitude: SCR and stigma. Income (β = 0.24, *p* = 0.036) and SCPR (β = 0.40, *p* = 0.001) contributed positively to wellbeing, whereas SCNR contributed negatively to wellbeing (β = −0.56, *p* = 0.001). Stigma made no contribution to wellbeing. This step contributed 15% to the explained variance. In the final model, we inserted the personal and social resources of self-esteem (β = 0.39, *p* = 0.001) and sense of community (β = 0.31, *p* = 0.002), which contributed positively to wellbeing. SCNR contributed negatively to wellbeing (β = −0.26, *p* = 0.046), whereas age, income, stigma, and SCPR made no contribution to life satisfaction. This step contributed 22% to the explained variance. The percentage of the explained variance of wellbeing by the regression analyses was 45%.

**TABLE 2:** Beta coefficients of hierarchical regressions of demographic variables, community’s attitude and perceptions and personal and social resources to wellbeing.

Measures	Step I			Step II			Step IV		
	***B***	**β**	**SE**	***B***	**β**	**SE**	***B***	**β**	**SE**
Age	−0.01	−0.07	0.01	0.00	0.03	0.01	−0.01	−0.08	0.01
Income	0.15	0.31**	0.06	0.11	0.24*	0.05	0.08	0.16	0.05
Stigma				0.05	0.08	0.07	0.10	0.16	0.06
SCPR				0.29	0.40***	0.09	0.13	0.18	0.08
SCNR				−0.34	−0.56***	0.08	−0.16	−0.26*	0.08
Self-esteem							0.39	0.39***	0.08
Sense of community							0.19	0.30**	0.06
*R* ^2^	0.08*			0.23***			0.45***		
Δ *R*^2^	0.08*			0.15***			0.22***		

*p < 0.05, **p < 0.01, ***p < 0.001.

### Path analysis modeling: examining the option of a mediation model

An indirect effects analysis was conducted using Hayes’ PROCESS macro (2022) in SPSS. The bootstrap procedure was used to further evaluate the significance of the mediator. We based the estimate of the indirect effect by running 1,000 bootstrap iterations of computed samples and used a 95% confidence interval (CI). The exogenous variables were those that represented the community’s attitude and perception as well as the background variables that made a significant contribution to wellbeing in the regression analysis: income, stigma, and SCR. The endogenous variables were self-esteem and sense of community. The target variable was wellbeing.

**FIGURE 1:**
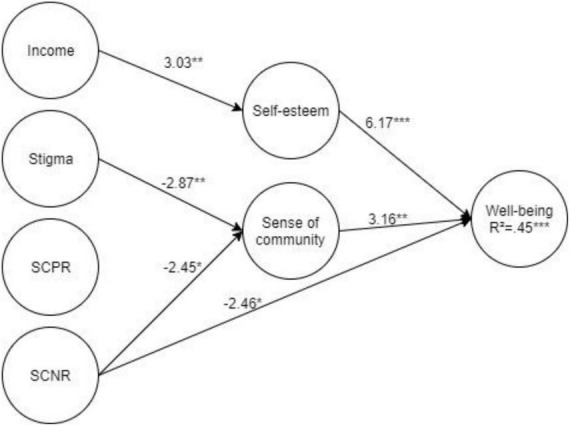
Standardized results of the path analysis modeling of the effects of demographic variables, community’s attitude and perceptions, and personal and social resources on wellbeing. *p < 0.05, **p < 0.01, ***p < 0.001.

Figure 1 presents the β coefficients of the direct effects found to be significant in the mediational model (*p* < 0.05 and lower). Table 3 presents the indirect effects. The findings point to a positive indirect (β = 0.12) correlation via self-esteem between income and wellbeing. Stigma made a negative indirect (β = −0.07) contribution via sense of community to wellbeing. SCNR made a negative direct (β = −2.46) and indirect (β = −0.07) contribution via sense of community to wellbeing. SCPR, however, made no contribution to wellbeing.

**TABLE 3:** Betas and SEs of the indirect effect of the path analysis modeling.

Independent	Mediator	Dependent	Independent to mediator	Mediator to dependent	Direct effect	Indirect effect
Income	Self-esteem	Wellbeing	3.03** (0.08)	6.17*** (0.11)	1.25 (0.03)	0.12* (0.04)
Stigma	Sense of community	Wellbeing	−2.87** (0.09)	3.16*** (0.11)	0.80 (0.06)	−0.07* (0.03)
SCNR	Sense of community	Wellbeing	−2.45* (0.11)	3.16*** (0.11)	−2.46* (0.06)	−0.07* (0.04)

*p < 0.05, **p < 0.01, ***p < 0.001.

## Discussion

The current study focused on the implications of community attitudes for the wellbeing of community members of religious minorities who deviate from the norms of their community. The findings reveal the complex situation of Ultraorthodox young men in Israel who enlist in the army. On the one hand, the community’s attitude toward these young men creates a risk situation for their wellbeing. On the other hand, their self-esteem and the sense of community they feel with the Ultraorthodox community were found to be significant protective factors for their wellbeing. To the best of our knowledge, the current study is the first to investigate the wellbeing of young men from the Ultraorthodox community who served in the Israeli army.

In the current study we investigated the contribution of self-esteem (a personal resource) and sense of community (a communal resource) to the young men’s wellbeing. Both resources were found to be protective factors for the young men’s wellbeing, as well as mediators in the correlation between income and wellbeing, and the community’s attitude and wellbeing. Regarding self-esteem, the positive contribution of this resource to wellbeing has been investigated thoroughly among at-risk populations (e.g., Çakar and Tagay, 2017; Fuentes et al., 2020), and specifically among at-risk Ultraorthodox youth (Itzhaki et al., 2018a). The novel finding of the current study, however, was the mediating role of self-esteem in the association between income and wellbeing. Income is generally considered to be a protective factor for wellbeing among at-risk populations. For example, among care leavers, income is a key factor for their success in the transition to adulthood, given that when they have money, they have the ability to manage life on their own (Benbenishty and Magnus, 2008). However, in the current study, income was not found to make a direct contribution to wellbeing among the study participants. Income made only an indirect contribution to wellbeing via self-esteem. A possible explanation for this finding might lie in the specific characteristics that typify the Ultraorthodox community. The Ultraorthodox community is considered to be “poor by choice” – namely, the men in this community are expected to learn Jewish subjects and not to work. As a result, the Ultraorthodox community is one of the poorest sectors in Israel, with a poverty rate of more than 50% (Kliner-Kasir and Tsachor-Shai, 2017). It could be that this way of life, in which people are meant to be content with little in the way of material goods, influences the way money is viewed. In the Ultraorthodox community money is thought to be only a means to an end (i.e., to exist), not something toward which one aspires. Thus, no direct correlation was found between income and wellbeing. However, in a community that sanctifies those who sit and learn Jewish subjects, those who fail to do so might need to adopt an alternative way to define themselves as successful. Thus, having a high income may have become the definition of success for those who could not succeed in accordance with the communal norms: Namely, high levels of income contributed to higher levels of self-esteem, which in turn contributed to higher levels of wellbeing.

The positive contribution of sense of community to wellbeing has been found in many studies (Stewart and Townley, 2020). However, the positive contribution that was found in the current study is a novel finding given that the study population violated the communal norms and had become part of a community consisting of soldiers, within the IDF – a community that the Ultraorthodox community finds objectionable. A reasonable result of this process could have been that the young men’s feeling of sense of community with the Ultraorthodox community would no longer be a protective factor for their wellbeing. The findings revealed how these young men wished to feel and be part of the Ultraorthodox community despite the choice they made to enlist. It could be that this group, by remaining connected to the Ultraorthodox community, is trying, perhaps not even consciously, to create a legitimate new group in the Ultraorthodox community, of youth who enlist in the army and remain an integral part of the Ultraorthodox community.

However, the community’s attitude, which was investigated in the current study via stigma and SCR, highlights the fact that legitimizing military service in the Ultraorthodox community will take time. Surprisingly, stigma made no direct contribution to the young men’s wellbeing. However, it made a negative indirect contribution via sense of community. Link and Phelan (2001) explained that the existence of multiple stigma mechanisms and multiple stigma outcomes helps explain why members of stigmatized groups are disadvantaged in a broad range of life domains such as psychological wellbeing. According to their model, stigma involves the loss of status and a downward placement in the status hierarchy. The findings of the current study suggest that this loss of status was expressed in the correlation between higher levels of stigma and lower levels of sense of community. In this way, stigma impairs the effect of sense of community as a protective factor for these young men’s wellbeing.

Together with stigma, these young men also had to cope with SCNR. Consistent with previous studies (Itzhaki et al., 2018b, 2019), SCNR was found to make a negative contribution to wellbeing both directly and indirectly via sense of community. As feelings of autonomy are considered to be a key factor in one’s successful transition to adulthood (Melendro et al., 2020), SCNR, which suppresses autonomy, is a major risk factor for these young men’s wellbeing. Moreover, the indirect contribution highlights the effect of the community members’ negative attitude toward these young men, which not only impairs their wellbeing but also impairs their sense of community. It seems that for the young men themselves, there is no contradiction between their military service and their feeling of belonging to the Ultraorthodox community. However, the findings of the current study show that what impairs their sense of community is the negative attitude they receive from community members. Moreover, both SCNR and stigma were found to be negatively correlated with self-esteem. The negative relationships between SCNR and stigma, and between self-esteem, sense of community, and wellbeing, emphasize how significantly the community members’ negative attitude toward the participants curtailed their life options (Link and Phelan, 2001).

In line with our study goal, our findings emphasize the socio-psychological implications of deviating from the norms of religious minority communities. Indeed, our findings reveal the process by which the wellbeing of those who deviate from norms becomes impaired. Based on self-determination theory (Ryan and Deci, 2000), it seems that in its reaction to the violation of norms, the religious community damages the three basic needs emphasized by the theory. The need for autonomy is harmed by the use of SCR, the need for relatedness is harmed by an impaired sense of community, and the need for competence is harmed by impaired self-esteem. These three basic needs are crucial for the individual’s wellbeing (Ryan and Deci, 2000), and our study demonstrates the way in which the communal attitude toward those who violate communal norms harms these needs and contributes to lower levels of wellbeing. The implication of this theoretical understanding is that individuals from religious minorities who want to be part of the religious community, but cannot, or do not want to adhere to communal norms, will experience an impairment in their wellbeing in accordance with community members’ attitude toward them. These theoretical implications shed light on the risk situation of those who deviate from norms in religious minority communities.

A surprising finding of the current study was the role of SCPR in the contribution to wellbeing. Based on previous studies, which found SCPR to be a contributing factor to positive psychological outcomes (Itzhaki et al., 2018a; Itzhaki and Cnaan, 2019), we hypothesized that SCPR would positively contribute to wellbeing. This assumption was confirmed in the second level of the regression process, but when we inserted the personal and social resources, the contribution of SCPR disappeared. Moreover, there was no contribution of SCPR in the mediation model. A possible explanation for this finding could be the differences between the study populations in the different studies. In the aforementioned previous studies in which a positive contribution of SCPR was found, the study participants were groups whose status in the community could be changed depending on whether, they behave in accordance with communal expectations: dropout Ultraorthodox youth (Itzhaki et al., 2018a) and Orthodox congregation members (Itzhaki and Cnaan, 2019). However, the population of the current study were seen as deviating from norms due to their enlisting in the army and wearing the army uniform: obvious and visible signs of defying a community norm. Thus, the contribution of the positive attitude of the community as expressed in SCPR was limited, and not expressed in the presence of personal and social resources. These findings are in line with the findings of a study on SCR among Muslim divorced women, in which there was no contribution of SCPR to wellbeing. In this case as well, the positive attitude of the Muslim community toward divorced women who behaved in accordance with the community’s expectations did not change the fact that they were seen as “deviants” who violated the communal norm of maintaining the wholeness of the family (Abu ras, 2021).

It is worth mentioning that the fact that age made no contribution to wellbeing highlights the complex situation of these Ultraorthodox young men (that is, unlike other Israelis, they are not required to enlist after high school, and therefore their enlistment occurs across a range of ages). Most of these young men were in the transition-to-adulthood phase, which is a complicated phase in general, and for at-risk populations, it entails additional challenges. Diminishing the protective factors for their wellbeing can put them at heightened risk during this complex stage in their lives.

### Limitations and future studies

The study’s limitations included challenges in getting the target population to participate in the research, a challenge that required our use of non-random sampling. This kind of sampling can hinder the ability to generalize from the findings, even though most quantitative research studies utilize non-random samples. In future studies, we would recommend using other/additional kinds of sampling (Onwuegbuzie and Collins, 2007). Another limitation is that the current study was cross-sectional; namely, we found correlations but could not establish causality. A longitudinal study should be conducted to examine how the model works over time.

Furthermore, in this study we focused on a particular minority group in Israeli society, but it would be valuable for future researchers to examine the effects of community members’ attitudes toward norm violators in other religious minority communities. For example, community attitudes may make a significant contribution to various psychological factors in the case of drug and alcohol addiction among Amish and Islamic sects (Cates and Weber, 2013; Al Ghaferi et al., 2017; Mendez Ruiz, 2017; Badanta et al., 2020) or among divorced women in the Christian community (Afifi et al., 2013). Understanding the experiences of minority communities and the impact of communal attitudes toward norm violators can help professionals in the field develop effective interventions and support systems for those individuals facing psychological challenges.

Finally, our findings are based on the participants’ self-reports. Although self-reports in other studies on at-risk populations have been found reliable, stronger evidence can be achieved in the future by integrating other forms of data, such as the perceptions of parents, spiritual leaders, and community members.

### Implications for practice

From a practical point of view, the findings of the present study point to the great influence of the community’s negative attitude toward young men from the Ultraorthodox community who enlist in the army. Thus, we have two main recommendations. The first concerns professionals who treat and assist these young men both during their military service and afterward. We believe that professionals should develop intervention programs that enhance these young men’s self-esteem. Moreover, as income was found to be a contributing factor to self-esteem, preparatory programs for the world of work must be developed. These programs should be adapted to Ultraorthodox society. Given that Ultraorthodox boys do not study specific subjects (e.g., math and English) in yeshiva, their ability to integrate into the Israeli free market after they have served in the army is more limited, and preparatory programs should be tailored specifically to them. In parallel, professionals should encourage these young men to complete the studies they did not complete previously, and direct them to sources of assistance around completing studies and matriculation.

Our second recommendation concerns the connection between the Israeli army and the Ultraorthodox community, especially this community’s spiritual leadership. We believe that the use of stigma and SCNR toward young men from the Ultraorthodox community who enlist helps this community send a message to its members – that is, that enlisting in the army is forbidden, or at the very least greatly frowned upon. However, as learning in a yeshiva from morning till night does not suit all young men, the army can be seen as an alternative framework that will prevent these young men from succumbing to loitering, crime, drugs, and other risky behaviors. Thus, we would recommend that the army think together along with spiritual leaders and professionals about ways to keep Ultraorthodox soldiers close to the Ultraorthodox community. It is clear that as long as they serve in the army and wear the IDF uniform, it will be difficult to do so. However, a change in policy, according to which contact is maintained with them throughout their military service, may help in reducing the communal stigma and the SCNR they experience, which in turn would increase their wellbeing. This new approach might make it easier for them to return to the community when they finish their service, and to successfully integrate into adult lives.

## Data availability statement

The raw data supporting the conclusions of this article will be made available by the authors, without undue reservation.

## Ethics statement

The studies involving human participants were reviewed and approved by the Tel Aviv University IRB-04750. The patients/participants provided their written informed consent to participate in this study.

## Author contributions

NM-Y and YI-B contributed to the conception and design, and writing of the work. SS-R organized the database. All authors contributed to manuscript revision, read, and approved the submitted version.
